# IMPROVED AGE- AND GENDER-SPECIFIC RADIATION RISK MODELS APPLIED ON COHORTS OF SWEDISH PATIENTS

**DOI:** 10.1093/rpd/ncab075

**Published:** 2021-05-29

**Authors:** Martin Andersson, Keith Eckerman, David Pawel, Anja Almén, Sören Mattsson

**Affiliations:** Department of Radiation Physics, Institute of Clinical Sciences, Sahlgrenska Cancer Center, Sahlgrenska Academy, University of Gothenburg, Gothenburg, Sweden; Medical Radiation Physics, Department of Translational Medicine, Lund University, Skåne University Hospital, SUS, Malmö, Sweden; Center for Radiation Protection Knowledge, Oak Ridge National Laboratory, Oak Ridge, TN, USA; United States Environmental Protection Agency, Washington, DC, USA; Medical Radiation Physics, Department of Translational Medicine, Lund University, Skåne University Hospital, SUS, Malmö, Sweden; Department of Radiation Protection, Swedish Radiation Safety Authority, Stockholm, Sweden; Medical Radiation Physics, Department of Translational Medicine, Lund University, Skåne University Hospital, SUS, Malmö, Sweden

## Abstract

The aim of this study is to implement lifetime attributable risk (LAR) predictions for radiation induced cancers for Swedish cohorts of patients of various age and sex, undergoing diagnostic investigations by nuclear medicine methods. **Methods:** Calculations are performed on Swedish groups of patients with Paget's disease and with bone metastases from prostatic cancer and diagnosed with bone scintigraphy with an administration of 500 MBq ^99m^Tc-phosphonate. **Results:** The inclusion of patient survival rates into the calculations lowers the induced radiation cancer risk, as it takes into account that cohorts of patients have shorter predicted survival times than the general population. **Conclusion:** LAR estimations could be valuable for referring physicians, nuclear medicine physicians, nurses, medical physicists, radiologists, and oncologists and as well as ethical committees for risk estimates for specific subgroups of patients. Caution is however advised with respect to application of LAR predictions to individuals (because of individual sensitivities, circumstances, etc.).

## INRODUCTION

In all radiological diagnostics, it is important that practitioners, referrers, physicians, nurses and medical physicist in various imaging disciplines and others in healthcare are aware of how much radiation a patient may receive from a medical procedure and the associated health risk. It is also important that the professional informs patients and/or their representatives of the advantages and disadvantages of specific investigations. In diagnostic, nuclear medicine seldom results in tissue effects, with the exception of possible high skin doses for severe cases of extravasation. In all irradiated tissues, which are not killed by radiation, there is a risk of stochastic effects in the form of increased risk of cancer during the rest of the life and of hereditary effects if gonads are exposed. Although ionising radiation is a comparatively weak carcinogenic agent, it is still necessary to be aware of the risk of cancer induction in investigated patients as well as other risks connected to the investigation. This should be done to balance risks of performing versus not performing the investigation. The need to estimate and communicate risks in connection with medical use of ionising radiation is highlighted in the European Union directive (2013/59/EURATOM 2014), which states that, ‘Member States shall ensure … wherever practicable and prior to the exposure taking place … ensures that the patient or their representative is provided with adequate information relating to the benefits and risks associated with the radiation dose from the medical exposure’.^(1)^

The most common way to express harm (mainly cancer during the rest of the individuals’ life) in relation to low doses of ionising radiation is to use the quantity effective dose (E). Effective dose is, however, not intended to provide risk estimates for medical exposures. Its purpose is to optimise conditions for radiation workers (18–65 years) or to estimate the risk for stochastic effects in the general public—groups with different age distributions than patients^(2)^. The effective dose is based on estimates of excess cancer incidence risk. The excess cancer incidence risk is then multiplied with a ‘lethality fraction’, a ‘nominal risk adjusted for lethality and quality of life’ and a ‘relative cancer free life lost’ fraction. The organ/tissue weighting factors are assessed and risk coefficients 5.5% per Sv for whole population and 4.1% per Sv for radiation workers are established. The effective dose is a robust unit for a normal population, but do not take age and sex into account. The reason for this, stated by the International Commission on Radiological Protection (ICRP), is that the system of protection should be sufficiently simple and robust^(2, 3)^.

Another approach for risk estimates is to use the lifetime attributable risk (LAR). LAR has been applied by the US National Research Council Committee to Assess Health Risks from Exposure to Low Levels of Ionizing Radiation in their report biological effects of ionizing radiation (BEIR) VII^(4)^. LAR describes excess deaths or disease cases over a follow-up period with population background rates determined by the experience of unexposed individuals. For the US population, the general baseline cancer risk is 39.3 and 37.7% for men and women, respectively,^(5)^ and LAR estimates the excess cancer risk from this baseline due to exposure of radiation. LAR assessments can also be made to estimate the radiation-induced mortality risk of a radiation exposure, by adding population-based cancer mortality data to the cancer morbidity predictions. The LAR calculations are based on three different variables, sex, attained age and age at exposure, to predict the risk to different radiosensitive organs and tissues. The US Environmental Protection Agency (EPA) made a number of extensions (e.g. risk estimates for alpha particles) and modifications (e.g. more extensive analysis of uncertainties associated with the radiogenic risk estimates) to the BEIR VII approach^(6)^. The EPA published LAR coefficients for radiation-induced cancer for 14 specific cancer sites and a category called ‘residual site’. The residual site cancers include cancers for which there were insufficient data from the Life Span Study of Japanese Atomic Bomb Survivors or other epidemiological studies to reliably quantify radiogenic site-specific risks. All EPA predictions were made for the US population and therefore based on data of US cancer statistics and life expectancy.

Both the ICRP and the EPA base the risk estimations mainly on data from the Life Span Study of A-bomb survivors. The risk models are based on the linear no threshold model and a dose and dose rate effectiveness factor to extrapolate risk data from levels of exposures. However, the LAR predictions have a clearer endpoint for the risk estimations compared with the concept of effective dose, and this risk model assesses both the excess cancer incidence and the premature cancer death.

**Figure 1:**
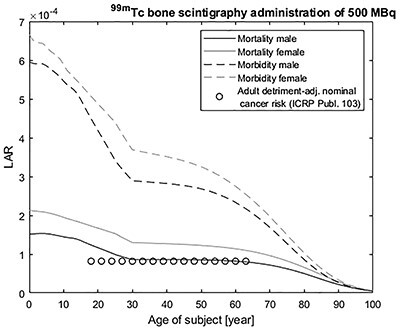
Age at exposure and sex-dependent cancer morbidity and mortality risks from an intravenously administered ^99m^Tc-phosphonates for bone scintigraphy using recommended age-dependent administrations^(7)^.

In previous studies,^(7, 8)^ we assessed the differences in outcome between effective dose and the published LAR coefficients of EPA for a ^99m^Tc bone scintigraphy, for ages at exposure between 0 and 110 years. The adjusted effective dose and the LAR values for mortality risks for adult males are comparable, as shown in Figure 1. This is expected as the detriment of effective dose is most similar to the radiation-induced risk of lethal cancer. However, the effective dose-based risk for adults (4.1% per Sv) of all ages and sex could also be misguided and difficult to explain. Due to the clearer endpoint and the three parameters, LAR method gives more data, which could be used in nuclear medicine.

### Administration versus acquisition time in nuclear medicine

In medical imaging, the primary goal is to produce images of clinically useful quality for the individual patient. When using X-rays or radiopharmaceuticals, a second goal is to use the lowest possible radiation dose to get this image quality. Therefore, the management of patient dose in nuclear medicine involves assessment of patient dose as well as image quality. For clinical measurements, there is a ‘time window’ when there are possibilities to evaluate image quality and extract the relevant information. Dose estimations need to be based on estimates of the biokinetic behaviour for the entire time the radionuclide is present in the body. The main task is to find the optimal relation between image quality/diagnostic efficacy and radiation dose for the individual patient. A feature of many nuclear medicine procedures is that for a certain administered activity, one can choose the acquisition time within a considerable range. Once the time window of a nuclear medicine investigation and the amount of radiation needed to achieve an acceptable image quality are determined, an age- and sex-specific LAR can be estimated, as shown in Figure 1. The LAR calculations give a possibility to change the administered activity based on sex and patient age to reach the same risk for all patient groups.

The LAR predictions published by the US EPA are all based on statistics for the US population and follow the general life expectancy. The aim of this study is to recalculate the LAR coefficients for diagnostic examinations of bone scintigraphy, based on adjustments to Swedish cancer statistic and survival data to account for the often shorter life expectancy for patient groups of Paget’s disease and for patient groups of prostate cancer with bone metastases.

## METHOD

The LAR assessments for each of the 15 specific cancer site are presented in Equation 1. The calculations are performed in three steps. The first step is to calculate for each cancer type, the age-specific excess rate of cancer diagnosis, $M\big(D,e,a\big)$. The $M\big(D,e,a\big)$ is a function of three variables, the absorbed dose (*D*) of the specific organ, the age (*e*) at the exposure and the attained age (*a*) of cancer diagnosis. The next step is to multiply the excess cancer rates by the probability of being alive at age *a*, $S(a)$ each year after the exposure, normalised by the probability of being alive at exposure. Finally, the LAR is obtained by integrating the adjusted excess cancer rates over attained age. The integration starts 5 years (two for leukaemia) after the exposure.

(1) \begin{align*} \mathrm{LAR}{\left(D,e\right)}_{\mathrm{Sex}}={\int}_{e+L}^{110}M\left(D,e,a\right)\bullet \frac{S(a)}{S\left(e+L\right)}\mathrm{da} \end{align*}

where $D$ is the absorbed dose, $e$ is the age (year) at exposure, $L$ is the latency period (year) after exposure for which stochastic effects occurs, $a$ is the attained age (year), $S(a)$ and $S(e)$ are the survival rate at age *a* and *e*, respectively.

### Calculation of LAR for cohorts of Swedish patients

**Figure 2:**
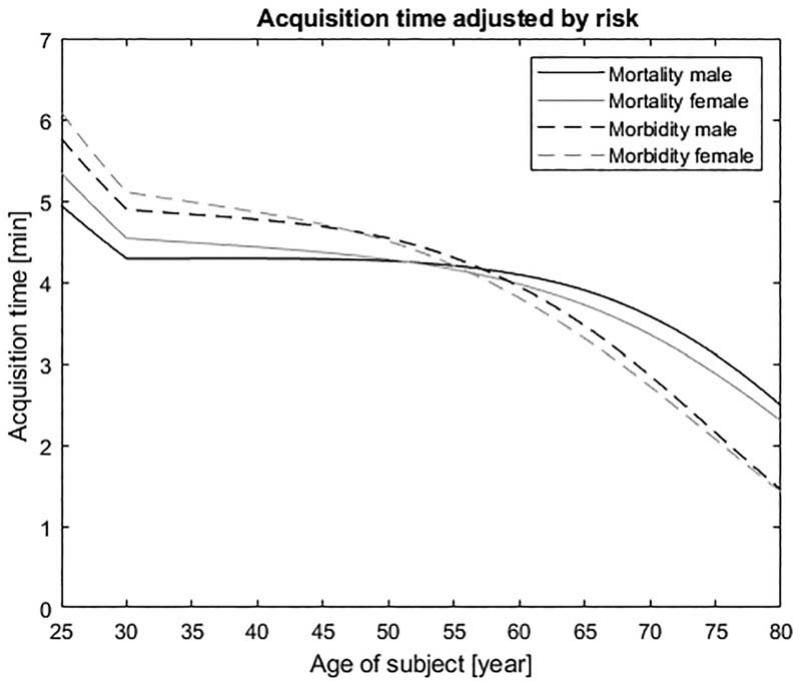
Age- and sex-specific acquisition times for an intravenously administered ^99m^Tc-phosphonates for bone scintigraphy, by adjusting the administered activity to get the same radiation risk for all ages.

The LAR predictions published by the EPA^(6)^ were recalculated for a Swedish population, based on survival rates from the Swedish National Board of Health and Welfare^(9)^ and cancer statistics from NORDCAN^(10)^. It should also be noted that cancer risk projections shown in Figure 2 for environmental exposures are based on survival functions for normal populations. However, patients with Paget’s disease have a 5-year survival rate of 93%^(11)^. For prostate cancer with bone metastases, the 2-year survival rate is 68% for one lesion and 22% for more lesions^(12)^. An additional parameter $R\big(t,a\big)$ is included to account for these shorter age- and sex-specific life expectancies. The probability of survival rate for age $a$ is calculated as the number of persons alive at year $a$ divided by the initial persons alive at age 0. The patient-specific survival rate, $S{(a)}_{\mathrm{Pat}.\mathrm{Spec}}$, for, e.g. Paget’s disease is modified for the first 5 years after exposure as

$$\begin{eqnarray*} && S\left(a,R\left(t,a\right)\right) \\ && = \frac{N{(a)}^{\ast }}{N(0)}=\frac{N(a)\ast \mathrm{ASP}{\left(R\left(t,a\right)\right)}_{\textrm{Paget}^{\prime}\textrm{s} \textrm{disease}}}{N(0)} \end{eqnarray*}$$

where $N(a)$ and $N(0)$ are the total number of healthy persons of a specific gender and alive at age $a$ and 0. $N{(a)}^{\ast }$ is the total number of patients living at age $a$ generated by multiplying $N(a)$ with the annual survival probability, ASP$\big(R\big(t,a\big)\big)$, of that specific cohort.

### ^99m^Tc bone scintigraphy

Bone scintigraphy with phosphate analogues labelled with ^99m^Tc is used to evaluate the distribution of active bone formation in the skeleton related to malignant and benign diseases. A biokinetic model and absorbed doses to organs and tissue for ^99m^Tc-labelled phosphates and phosphonates are presented for five different ages 1, 5, 10, 15 and adult (≥25 years) in ICRP publication 128^(13)^. In general, the reference phantom representing a 20-year-old person except for the bone composition, and therefore for bone-seeking radiopharmaceuticals, the age-dependent interpolation is performed up to 25 years. The European Association of Nuclear Medicine (EANM) guidelines for bone scintigraphy of adults with ^99m^Tc recommend an average activity administered by a single intravenous injection of 500 MBq for all adults^(14)^. An example on how the LAR values could be implemented in optimisation of nuclear medicine is in the modification of acquisition times; the EANM guidelines for ^99m^Tc-labelled phosphonate are an acquisition time of 3–5 min. In this calculations, the reference acquisition time was set to 4 min. The reference administration was set to 500 MBq, and from this administration, age-specific LAR risk values were calculated. From these calculations, an average cancer risk for all ages was determined. Since the risk is proportional to administration and administration is inversely proportional to the acquisition time, an age-specific acquisition time could be determined.

## RESULTS AND DISCUSSION

The effect of the modification of acquisition time for ^99m^Tc-labelled bisphosphonate is shown in Figure 2. The figure shows that, as the life expectancy of older patients is shorter, the lifetime cancer risk will lower, which corresponds to a justification of administered higher activity and compensate this with a shorter acquisition time without altering the diagnostic quality of the image. This figure shows an example on how the LAR values could be used in the clinic, e.g. if older patients have difficulty to be in a fixed position on the camera table during the whole procedure. Making the LAR values an extra parameter of freedom might be of importance in optimising the medical procedures.

**Figure 3:**
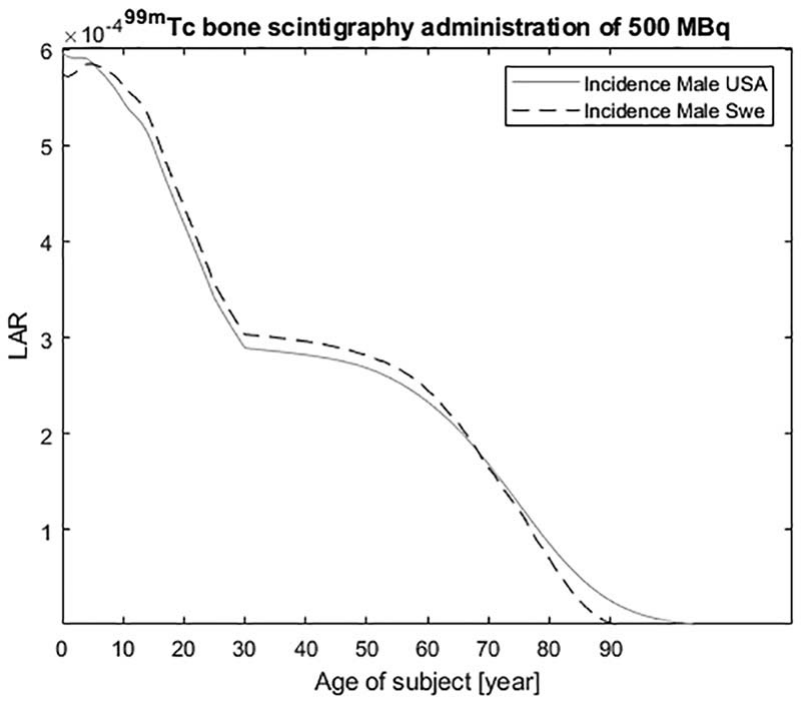
LAR incidence as function of the age at exposure for a Swedish male population based on the last 30 years of Swedish cancer statistic^(9)^ and a US male population, both based on the risk assessments given by US EPA^(6)^ for an intravenously administered ^99m^Tc-phosphonates for bone scintigraphy of 500 MBq.

A comparison between the LAR incidence for bone scintigraphy with an administration of 500 MBq ^99m^Tc is shown in Figure 3. The Swedish male population, is based on survival rates from the Swedish National Board of Health and Welfare^(9)^ and the last 30 years of Swedish cancer statistic from NORDCAN^(10)^ and for a US population is shown in Figure 2. Both estimates are based on risk assessments given by EPA^(6)^. The predictions for both populations are close to each other. There is a slighter higher risk for newborn, children and adolescents of the Swedish male population than for similar US groups. This is probably due to differences in cancer statistics. For adult and senior (60+ years), the risk values are lower for the Swedish population than for the US populations.

**Figure 4:**
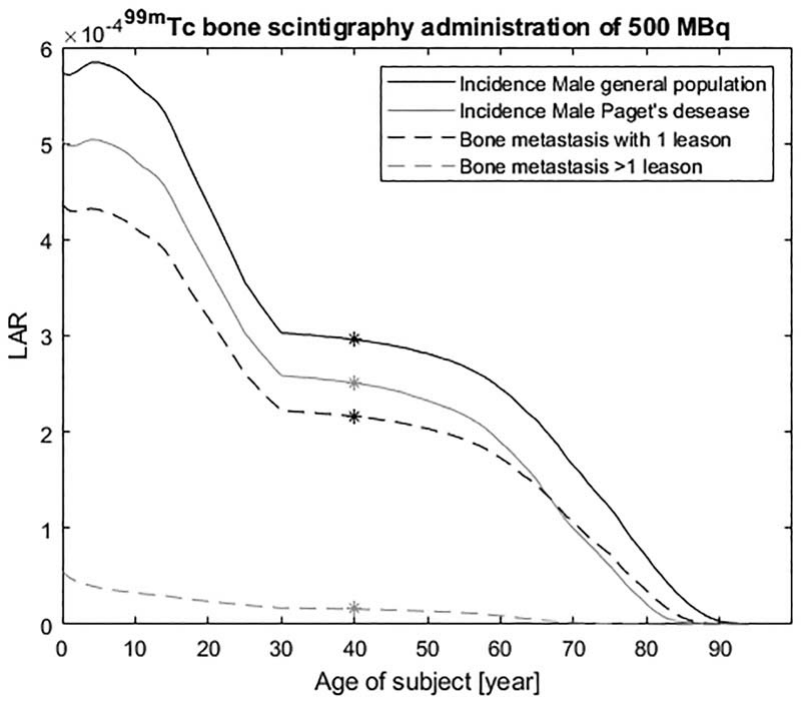
LAR incidence as function of the age at exposure for a normal Swedish male population and for three different groups of Swedish male cancer patients (Paget’s disease, and prostate cancer with one bone metastases with one lesion or more than one) given an intravenously administered ^99m^Tc-phosphonates for bone scintigraphy of 500 MBq.

In Figure 4, LAR predictions are shown for a bone scintigraphy with an administration of 500 MBq ^99m^Tc, applied on a normal Swedish male population and for three different groups of Swedish male patients. The LAR risks are the risks of receiving a cancer, incidence, for a cohort of Paget’s disease, and prostate cancer with bone metastases of one lesion and >1 lesions are estimated. For the normal Swedish 40-year-old male population, the cancer incidence risk is 0.00030 (or 1 in 3400) and the corresponding risks are for Paget’s disease: 0.00025 (or 1 in 4000), bone metastasis with one lesion 0.00022 (or 1 in 4600), bone metastasis >1 lesions 0.000016 (or 1 in 63 000), respectively.

The result can be interpreted in two ways. For male patients with prostate cancer with bone metastases of >1 lesions have a low probability of developing a cancer later in life, e.g. ~20 times lower risk for a 40 years old. Additional diagnostic imaging would have a low impact on the cancer risk. The drawback is that in this case the number of metastases is probably unknown before the procedure. However, this still gives a level of risk and further procedures can be decided from the gained information. For cohorts with a higher survival probability, e.g. as in case of Paget’s disease, the cohort group only differ 15% from the general population, this should also reflect the diagnostic protocols of this patient group and the optimisation of protocols should also include the probability of radiation-induced cancer incidence.

If a radiological examination is clinically motivated, its benefits will almost always far outweigh the radiation risks. In spite of that, there is a need to optimise the examinations to reduce the risk as much as possible. A common argument in medical imaging is that patient groups with low survival probability can be given a higher radiation absorbed dose, and in many cases, this might be true. This study provides examples of the possibility of quantifying the risk, and thus gain a better basis for such decisions and information. Specifically, in bone scintigraphy, it quantifies the radiation risk for, e.g. patients with prostate cancer with bone metastases and patients with Paget’s disease. This allows information to be included in the physician’s determination of diagnostic tools and in the information communicated to patients.

## FUNDING

This project was funded by Swedish Radiation Safety Authority (grant number: SSM2018-2163).

## DISCLAIMER

The views expressed in this article are those of the authors and do not necessarily represent the views or policies of their organisations.

## CONFLICT OF INTEREST

No conflict of interest was declared.

## References

[1] European Council Directive 2013/59/Euratom on basic safety standards for protection against the dangers arising from exposure to ionising radiation and repealing Directives 89/618/Euratom, 90/641/Euratom, 96/29/Euratom, 97/43/Euratom and 2003/122/Euratom. OJ of the EU. L13 57, 1–73 (2014).

[2] ICRP . The 2007 recommendations of the International Commission on Radiological Protection. ICRP Publication 103. Ann. ICRP 37(2–4), 1–332 (2007). 10.1016/j.icrp.2007.10.003 18082557

[3] Harrison, J. D., Balonov, M., Martin, C. J., Ortiz Lopez, P., Menzel, H.-G., Simmonds, J. R., Smith-Bindman, R. and Wakeford, R. Use of effective dose. Ann. ICRP 45(1S), 215–224 (2016). 26980800 10.1177/0146645316634566

[4] NRC . Health Risks from Exposure to Low Levels of Ionizing Radiation — BEIR VII. (Washington, DC: The National Academies Press) (2006). 25077203

[5] Simon, S. Facts & Figures 2019: US Cancer Death Rate has Dropped 27% in 25 Years. American Cancer Society ACS. Available on https://www.cancer.org/latest-news/facts-and-figures-2019.html (2019).

[6] EPA . Radiogenic Cancer Risk Models and Projections for the U.S. Population. (Washington, DC: EPA) 402–R–11–001. pp. 1–175 (2011).

[7] Andersson, M., Eckerman, K. and Mattsson, S. Lifetime attributable risk as an alternative to effective dose to describe the risk of cancer for patients in diagnostic and therapeutic nuclear medicine. Phys. Med. Biol. 62, 9177–9188 (2017). 29064376 10.1088/1361-6560/aa959c

[8] Andersson, M., Eckerman, K., Powel, D., Almén, A. and Mattsson, S. Improved radiation risk models applied to different patient groups in Sweden. Radiat. Gygiena 12(2), 44–54 (2019).

[9] Swedish National Board of Health and Welfare . The National Board of Health and Welfare's statistics database contains cause of death data. Available on http://www.socialstyrelsen.se/statistics/statisticaldatabase/help/causeofdeath (accessed on 20 May 2020).

[10] Engholm, G., et al. NORDCAN: Cancer Incidence, Mortality, Prevalence and Survival in the Nordic Countries, Version 8.1 (28.06.2018). Association of the Nordic Cancer Registries. Danish Cancer Society. Available on http://www.ancr.nu, (accessed on 20 May 2020).

[11] Van Staa, T. P., Selby, P., Leufkens, H. G. M., Lyles, K., Sprafka, J. M. and Cooper, C. Incidence and natural history of Paget’s disease of bone in England and Wales. J. Bone Miner. Res. 17(3), 465–471 (2002). 11878305 10.1359/jbmr.2002.17.3.465

[12] Yucel, B., Celasun, M. G., Oztoprak, B., Hasbek, Z., Bahar, S., Kacan, T., Bahceci, A. and Seker, M. M. The negative prognostic impact of bone metastasis with a tumor mass. Clinics 70, 535–540 (2015). 26247664 10.6061/clinics/2015(08)01 PMC4518841

[13] ICRP . Radiation dose to patients from radiopharmaceuticals: a compendium of current information related to frequently used substances. ICRP Publication 128. Ann. ICRP 44(2S), 1–321 (2015). 26069086 10.1177/0146645314558019

[14] Van den Wyngaert, T. et al. The EANM practice guidelines for bone scintigraphy. Eur. J. Nucl. Med. Mol. Imaging 43, 1723–1738 (2016). 27262701 10.1007/s00259-016-3415-4 PMC4932135

